# Medication safety curriculum: enhancing skills and changing behaviors

**DOI:** 10.1186/s12909-015-0521-0

**Published:** 2015-12-29

**Authors:** Kelly D. Karpa, Lindsay L. Hom, Paul Huffman, Erik B. Lehman, Vernon M. Chinchilli, Paul Haidet, Shou Ling Leong

**Affiliations:** 1 Department of Pharmacology, Pennsylvania State University College of Medicine, Mail Code R130, 500 University Dr., Hershey, PA 17033 USA; 2 Public Health Sciences, Pennsylvania State University College of Medicine, Hershey, PA USA; 3 General Internal Medicine, Pennsylvania State University College of Medicine, Hershey, PA USA; 4 Family and Community Medicine, Pennsylvania State University College of Medicine, Hershey, PA USA

**Keywords:** Inter-professional education, Medication safety, Medication reconciliation, Medication optimization, Patient-centered medical home, Clinical pharmacology, Longitudinal curriculum, Medical education, Value-added

## Abstract

**Background:**

Adverse drug reactions are a leading cause of death in the United States. Safe and effective management of complex medication regimens is a skill for which recent medical school graduates may be unprepared when they transition to residency. We wished to assess the impact of a medication safety curriculum on student competency when evaluating medication therapeutic appropriateness as well as evaluate students’ ability to transfer curricular material to management of patients in clinical settings.

**Methods:**

To prepare 3rd and 4th year medical students to critically evaluate medication safety and appropriateness, we developed a medication reconciliation/optimization curriculum and embedded it within a Patient-Centered Medical Home longitudinal elective. This curriculum is comprised of a medication reconciliation workshop, in-class and individual case-based assignments, and authentic patient encounters in which medication management skills are practiced and refined. Pre- and post-course competency and skills with medication reconciliation/optimization are evaluated by assessing student ability to identify and resolve medication-related problems (MRPs) in case-based assignments using paired difference tests. A group of students who had wished to enroll in the elective but whose schedule did not permit it, served as a comparison group.

**Results:**

Students completing the curriculum (*n =* 45) identified 75 % more MRPs in case assignments compared to baseline. No changes from baseline were apparent in the comparison group. Enrolled students were able to transfer their skills to the care of authentic patients; these students identified an average of 2.5 MRPs per patient from a panel of individuals that had recently transitioned from hospital to home. Moreover, patient questionnaires (before and several months following the medication encounters with assigned students) indicated that patients felt more knowledgeable about several medication parameters as a result of the student-led medication encounter. Patients also indicated that students helped them overcome barriers to medication adherence (e.g. cost, transportation, side effects).

**Conclusions:**

Novice learners may have difficulty transitioning from knowledge of basic pharmacology facts to application of that information in clinical practice. Our curriculum appears to bridge that gap in ways that may positively impact patient care.

## Background

Adverse drug reactions are cited as the fourth leading cause of death in the United States, accounting for $200 billion dollars in medical costs annually [1]. Patients are increasingly vulnerable to medication problems due to advancing age, complicated regimens, and polypharmacy. Other factors that contribute to adverse medication outcomes include inaccurate medication histories and prescribing by junior physicians [2]. Studies have shown that 10 % of prescriptions written by recent graduates contain an error, placing patients at risk for adverse drug events [3].

Prescribing is a complex task; it requires diagnostic skills, knowledge of clinical medicine, understanding of clinical pharmacology, communication skills, and critical judgment [3]. The transition, from being a medical student with a limited prescribing role to functioning as a junior resident with significant prescribing responsibilities, poses a steep learning curve [3]. New physicians consider prescribing to be the most difficult aspect of their job, and it is the component of “being a doctor” for which they feel least prepared [4]. When reflecting on their education, junior doctors indicate that insufficient emphasis was placed on the practical aspects of prescribing during their undergraduate curriculum, supporting the need to improve clinical pharmacology/therapeutics in medical curricula [5, 6].

The American Association of Medical Colleges (AAMC) issued Report X on *Education in Safe and Effective Prescribing Practices*, calling for medical schools to strengthen curricula pertaining to clinical pharmacology/therapeutics in 2008 [7]. The Liaison Committee on Medical Education (LCME) requires a basic science pharmacology curriculum, but there is no corresponding requirement for clinical pharmacology instruction. The National Board of Medical Examiners (NBME) does not currently report clinical pharmacology as a sub-score on the US Medical Licensing Step 2 exam. Strategies are needed to go beyond traditional pharmacology as *only* a basic science construct to curricula that help students learn to apply pharmacologic *principles* to the care of authentic patients before graduation.

To address this gap, we designed and implemented a medication reconciliation and optimization thread as a key component in a Patient Centered Medical Home (PCMH) longitudinal curriculum, a new elective at our institution. The medication-management component of the PCMH curriculum includes simulation with standardized patients, identification and correction of medication errors in clinical cases, and documentation of medication reconciliation (MR) and medication optimization (MO) in patients managed by students. We hypothesized that the clinical pharmacology components would improve student attitudes and confidence with medication management and enhance students’ clinical competency in identifying and correcting medication-related problems. Furthermore, we anticipated that, if the curriculum truly transformed students’ medication management behaviors, there would be a positive and discernible impact on patients as well.

## Methods

### The PCMH curriculum

A PCMH longitudinal elective was developed by a multi-disciplinary and inter-professional group of faculty (family physicians, internists and pediatricians, nurse practitioners, a pharmacist) and launched as an elective for 3rd and 4th year medical students. The PCMH elective was comprised of 4 workshops and 18 continuity clinical sessions at a designated ambulatory practice site; these sessions occurred longitudinally throughout the clinical years and were concurrent with core and elective clerkships. While at PCMH practice sites, students experienced continuity in their learning environment by working with one clinical preceptor and a small panel of patients. The longitudinal relationships that were established between students and their patients provided a context for students to practice principles learned during course workshops.

Medication reconciliation (MR) and medication optimization (MO) were topics of focus at course workshops. We defined MR as a formal process by which a complete and accurate *list* of medications is verified for accuracy. However, since reconciled medication lists can still include errors if drugs on the list are inappropriate for a given patient, we also encouraged students to think *critically* about the appropriateness of medications on a reconciled medication list. Thus, the terms “pharmacotherapeutic assessments” (PTA) and “medication optimization” (MO) were used interchangeably to define the process in which students were challenged to: critically examine drug lists to optimize medications (correct dosage, therapeutic monitoring, etc.) and assure appropriateness (consideration of comorbidities, allergies, timing, interactions, costs, etc.). The approach that students were encouraged to apply has been previously described and is included as a mnemonic in Table 1 [8].

**Table 1:** Mnemonic for MR: “CALL DOCT IF”

• Medications are the cause of patient’s current **C**hief complaint • Patient is experiencing **A**dverse effects caused by a medication • **L**ab values are abnormal because of a drug • **L**abs need to be monitored periodically because of a drug therapy • **D**osages/formulations are inappropriate for patient • Patient has a **D**iagnosis but lacks an appropriate medication • An appropriate **D**iagnosis is lacking for a prescribed medication • Therapeutic **D**uplication • Therapeutic **O**mission • Drug is **C**ontraindicated due to allergy or comorbidity • **T**ranscription error • **T**iming of medication administration is incorrect • Clinically-significant drug **I**nteractions • **F**inancial concerns due to medication costs	

### Student attitude and competency

We assessed student experiences and attitudes with MR and PTA before and after the course via a 32-item survey. In addition to demographic variables, the survey queried students about experiences and attitudes toward MR/PTA, perceived competency with performing medication assessments (identifying/resolving drug-related adverse events or therapeutic duplication/omission, appropriate monitoring of medications in individual patients), and ability to counsel patients on appropriate medication utilization. Seven-point rating scales for variables of interest ranged from 0 to 6 on a Likert-type scale with fixed terminal anchored responses.

During course workshops, key concepts pertaining to MR and PTA are facilitated by a pharmacist using active learning methods including a standardized patient simulated scenario and a series of clinical cases which have been previously described in detail [8, 9]. In addition, interactive classroom discussions highlight important principles pertaining to medication safety.

We assessed student competence in MR and PTA using clinical cases before the course (pre-course) as well as at the conclusion of the course (post-course). An example of one of our cases has been published previously [8]. Students were assigned to complete one of 20 clinical cases and were tasked with identifying medication-related problems and developing a reconciled medication list for the patient in the case vignette. Each clinical case consisted of a patient scenario and problem list, a medication list prior to hospitalization, in-hospital medications, hospital discharge medications, laboratory results, and additional information supplied by the patient (e.g. over-the-counter medications/dietary supplements, financial concerns, etc.). Students’ clinical case responses were graded based upon a percentage of medication-related problems identified per number of medication-problems built into the case. Students were not provided with feedback regarding their *pre*-course case assignment. Each clinical case contained at least 18 medication-related problems [8]. Each student completed the same clinical case again at the conclusion of the course, at which time personalized student feedback was provided to the student about the student’s pre-course and post-course responses.

### Clinical impact of student-initiated medication reconciliation

Students completed a MR Patient Project during their clinical sessions and performed MR and PTA with an actual patient that had been recently discharged from the hospital. Per the protocol approved by the Milton S. Hershey Medical Center Investigational Review Board, prior to interacting with the students, patients were given an explanation of research; those who were willing to participate in the student encounter completed a questionnaire in which they identified the number of medications taken, rated their own understanding of their medications, and indicated barriers to medication adherence. Completion of the MR Patient Project required students to reconcile the patient’s medications, provide medication education to the patient, and document any medication-related problems identified and resolved during the process. At the conclusion of the student encounter, patients/guardians who were willing to be contacted again provided their signature along with an address and/or phone number indicating approval for additional contact. For the patients who were amenable to be contacted again, the course director attempted to reach them 3 to 6 months later via telephone or a mailed questionnaire and asked them to rate the usefulness of the medication-education provided by the student and their current understanding of a variety of medication-related parameters.

### Comparison group

We recruited a comparison group of students who had wished to enroll in the PCMH elective, but whose schedules did not permit it. These students completed several of the same exercises as students enrolled in the PCMH course (MR/PTA questionnaire, MR clinical case); however, the comparison group did not participate in medication safety workshops or other PCMH course activities. The Institutional Review Board at the Milton S. Hershey Medical Center approved this study.

### Statistical analysis

Descriptive statistics were generated for all variables including medians and quartiles for continuous variables and frequency tables for categorical variables. Changes in pre- to post-course variables were compared within the enrolled and control groups using Wilcoxon signed-rank tests, whereas the enrolled and control groups were compared to each other via Wilcoxon rank-sum tests. An overall significance level of 0.05 was imposed, but a Bonferroni correction factor was applied to adjust p-values from the statistical tests of individual survey items. These analyses were performed in SAS Version 9.4.

## Results

### Student demographics

During the two years that the PCMH course has been offered, 45 students completed medication management course assignments. Two students opted to take the course in both their third and fourth years, thus maintaining continuity with their patient panel for 18 months. Of students who participated, the majority were women (Table 2). During the first academic year, all PCMH continuity sites were within Family Medicine practices; however, during subsequent years, sites included internal medicine (HIV and heart failure clinics; Veterans Affairs location), neurology (multiple sclerosis clinic), psychiatry (mood disorder clinic), and pediatrics (adolescent eating disorders clinic). The majority of enrolled students have opted for primary care residencies.

**Table 2:** Demographics of students enrolled in the PCMH elective

	First year course was offered	Second year course was offered	Totals
Total Students	13	34	47
Females	7	22	29
Males	6	12	18
3rd Year Students	3	28	31
4th Year Students	10	6	16
*PCMH Site:*			
Family Medicine	13	18	31
Internal Medicine	0	6	6
Pediatrics	0	2	2
Surgery	0	0	
Med-Peds	0	0	0
Neurology	0	4	4
OB/GYN	0	3	3
Psychiatry	0	1	1
*Residency Specialty* ^a^ *:*			
Family Medicine	6	9	15
Internal Medicine	3	6	9
Pediatrics	0	4	4
Surgery	0	2	2
Med-Peds	1	1	2
Neurology	1	1	2
OB/GYN	0	6	6
Psychiatry	0	2	2
Other Specialty	0	2	2

^a^Two students completed the course twice and one student took a medical leave of absence prior to commencement; therefore, there are three fewer students that matched to residencies compared to the overall number of students who were enrolled in the course

PCMH, Patient-Centered Medical Home; OB/GYN, obstetrics/gynecology; Med-Peds, medicine-pediatrics

Four 3rd year medical students who had not enrolled in the course served as a comparison group. All 4 students indicated a desire to learn more about PCMHs or had wished to enroll in the course, but were unable to participate due to scheduling conflicts. Of these students, three completed all pre- and post-course assessments; one was female and one selected a primary care residency.

### Pre-course and post-course abilities and confidence - student questionnaire

Completed MR/PTA questionnaires in which students rated their abilities and confidence performing medication-related critical thinking tasks before and after the course were available for 42 enrolled students and three comparison students. Prior to the course, there were no differences between the enrolled versus comparison students in any of the ability or confidence parameters assessed.

As shown in Table 3, students enrolled in the longitudinal course rated their ability to identify therapeutic duplication as well as evaluate medication appropriateness, counsel patients on appropriate medication use, and develop medication monitoring plans higher after the course. Similarly, these students also rated their confidence in reconciling medications higher after the course. No changes were observed in any parameter among the comparison group (data not shown). Although students enrolled in the course reported greater confidence in accurately optimizing medications after the course, this did not reach statistical significance after correction.

**Table 3:** Student reported abilities and confidence with reconciling and optimizing patients’ medications

Categories	Time	Enrolled median (1st quartile, 3rd quartile)	Unadjusted P-value	Adjusted P-Value
Ability				
Able to identify drug-related adverse effects	Pre	3 (2, 4)	0.01	NS
	Post	4 (3, 5)		
Able to correct drug-related adverse effects	Pre	2 (2, 4)	0.006	NS
	Post	3 (3, 4)		
Able to identify therapeutic duplication	Pre	3.5 (2, 4)	0.0002	0.005
	Post	5 (4, 5)		
Able to identify therapeutic omission	Pre	2 (2, 4)	0.002	NS
	Post	4 (3, 5)		
Able to evaluate medication appropriateness	Pre	3 (2, 4)	<0.0001	0.0026
	Post	4 (3, 5)		
Able to develop medication monitoring plans	Pre	3 (2, 3)	<0.0001	0.0026
	Post	4 (3, 5)		
Able to counsel patient on appropriate medication use	Pre	3 (2, 4)	<0.0001	0.0026
	Post	4 (4, 5)		
Confidence/comfort level				
Confidence to accurately perform MR	Pre	3 (2, 4)	0.001	0.026
	Post	4 (3, 4)		
Confidence to accurately perform PTA	Pre	2 (2, 3)	0.006	NS
	Post	3 (2, 4)		
PTA is challenging	Pre	5 (4, 5)	0.02	NS
	Post	4 (4, 5)		
Overwhelmed by PTA	Pre	4 (4, 5)	0.09	NS
	Post	4 (3, 5)		

Based on a 7-point Likert-type scale (0 to 6) with fixed terminal anchor responses

0 = less agreement with the statement

All values are represented as median (1st quartile, 3rd quartile). P-values represent comparison of enrolled student responses before and after the course

*MR* medication reconciliation, *PTA* pharmacotherapeutic assessments

### Students’ clinical competency with medication reconciliation

**Fig. 1:**
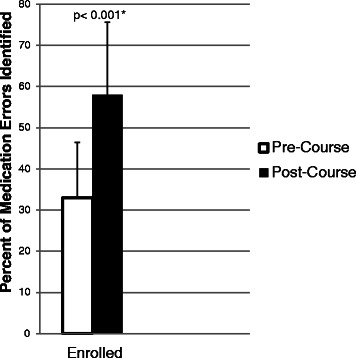
*n =* 44 enrolled students that completed both the pre-course and post-course Medication Reconciliation (MR) Clinical Case assignment

Forty-four enrolled students completed both a pre- and post-course MR clinical case that had medication problems intentionally incorporated. Prior to MR workshops, PCMH course students collectively identified 33 % of medication problems in the MR clinical cases, similar to the comparison group (which collectively identified 30 % of the medication problems; *P =* 0.95). After completing MR workshops and practicing MR/PTA with real patients during the year, enrolled students correctly identified 58 % of medication-related problems that were built into the clinical cases, a 75 % improvement over baseline (*P <* 0.0001, pre- to post-) (Fig. 1). In contrast, the comparison group did not perform any differently from baseline on the MR clinical cases at the end of the year (34 %; *P =* 0.5) suggesting that clinical exposure in traditional clerkships alone is insufficient to improve clinical competency with identifying medication-related problems.

### Impact of student-initiated MR on patients

Thirty-eight patients on students’ panels agreed to complete an initial questionnaire about their medication knowledge following a hospital discharge. The average age of these patients was 59 ± 20 years (range 5 to 90), and 25 (65 %) of these patients were female. The majority (*n =* 21) had completed high school and 10 individuals had also completed college or professional school. On average, these patients used 10.8 ± 4.8 prescription medications (range 4 to 21), 1.49 ± 1.5 (range 0 to 5) over-the-counter medications, and 0.81 ± 1.1 (range 0 to 4) dietary supplements. The average length of their recent hospital stay was 6.97 ± 5.69 days. Among these patients, students identified 95 medication problems (an average of 2.5 problems per patient) and corrected them with oversight of their clinical preceptor (Table 4).

**Table 4:** Medication-related problems identified by students during the medication reconciliation patient project

Type of medication problem	Number of patients impacted	Medications
Therapeutic Duplication	6	2 proton pump inhibitors (2), 2 statins, 2 beta blockers (3)
Therapeutic Omission at Discharge	8	Clopidogrel, Ramipril, Ranitidine, Vitamin B12 injection, Budesonide, Lisinopril, Tums, Prenatal vitamins, Carvedilol
Patient Did Not Take Medications Listed in Electronic Health Record (Non-Adherence)	11	Lisinopril, Bupropion, Aspirin, Multivitamin, Pantoprazole (2), Omeprazole, Metformin, Levothyroxine, Ergocalciferol, Pravastatin, Furosemide, Bactrim, Antipsychotic
Medications Used by Patients but Not Listed in Electronic Health Record	14	Fiber, Bisacodyl, Pantoprazole, Pravastatin, Magnesium, Vitamin B, Ciprofloxacin, Reservatol, CoQ-10, Ramipril, Ondansetron, Tylenol, Vitamin B-12, Tramadol, Aspirin, Glyburide, Ventolin, Multivitamin, Dexamethasone
Discharge List Included a Drug that Should Have Been Discontinued	1	Griseofulvin
Dosage too High	4	Warfarin, Aspirin, Oxycodone, Clonidine
Dosage too Low	9	Furosemide, Omeprazole, Doxycycline, Levothyroxine, Insulin, Hydrochlorothiazide, Gabapentin, Glimepiride, Lisinopril, Welchol
Drug-Disease Interaction	2	Omeprazole, Diclofenac
Drug-Drug Interaction	16	Amlodipine-simvastatin (2)
		Oxybutynin-potassium
		Escitalopram-pantoprazole
		Methotrexate-pantoprazole
		Sulfamethoxazole/trimethoprim-methotrexate
		Potassium-spironolactone
		Hydrochlorothiazide-vitamin D
		Hydrochlorothiazide -NSAIDs
		Aspirin-furosemide
		Fluconazole-citalopram
		Risperdone-ropinerole
		Warfarin (unspecified)
Currently Having an Adverse Drug Event	16	Omeprazole, Warfarin (3), Simvastatin, Metoprolol, Lasix, Amlodipine, Lisinopril, Insulin, Glimepiride, Antiretroviral, Clonidine, Tramadol, Tobramycin, Pravastatin, Hydrochlorothiazide, Medroxyprogesterone, Metformin, Risperidone
Lack of Indication for Medication	6	Omprepazole, Promethazine, Reservatol, Vitamin E, CoQ10, Wellbutrin, Cinnamon, Colchicine, Viagra
Previously documented allergy, intolerance, or side effect	1	Tenofovir
Taking brand when generic is available	1	Simvastatin

Out of the 38 patients who completed a questionnaire before medication reconciliation with a student, 14 patients (37 %) also completed a second questionnaire about their medication knowledge by phone or mail 3 to 6 months after the student interaction. Among those from whom we do not have a follow-up questionnaire, 10 patients specifically declined to be contacted with the second questionnaire, 1 patient expired, 3 questionnaires were returned due to change of address, and the remaining patients were mailed a follow-up questionnaire but did not return it.

For the patients who completed both the pre-MR questionnaire and the follow-up questionnaire, all indicated that they remembered speaking with a medical student about their medications, that the student was knowledgeable about medications, and that the student behaved in a professional manner. Seventy-five percent of the patients specifically recalled that the student had provided them with a list of all their medications. Forty-four percent indicated that as a result of the MR session with the student, they sought out additional medication-related information from another healthcare provider.

**Fig. 2:**
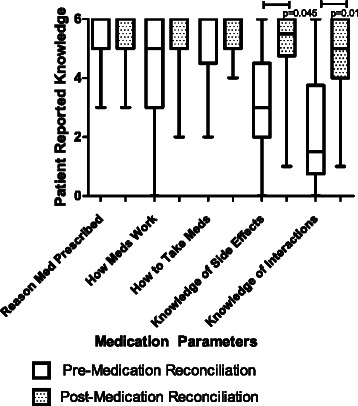
*n =* 14 patients who completed both the medication knowledge questionnaire prior to MR with students and the follow-up questionnaire. Patient self-reported knowledge of medication side effects and interactions remained significant after Bonferroni correction

Compared to patients’ self-described knowledge of their medications prior to the MR encounter with a student, at the time of follow-up, patients self-rated their own knowledge in the following medication domains higher: side effects caused by their medications [initial media*n =* 3 (2, 4.5) verus follow-up median 5.5 (4.75, 6.0); *P =* 0.045] and knowledge of potential drug-drug interactions [initial median 1.5 (0.75, 3.75) versus follow-up median 5.0 (4.0, 6.0); *P =* 0.002] (Fig. 2). No differences were detected over time for patients’ knowledge of why their medications had been prescribed, how the medications work, or how to take the medications.

After MR encounters with students, patients reported that students helped them overcome barriers to medication adherence. One patient reported that s/he overcame a cost barrier through a generic interchange, seven patients overcame transportation barriers by signing up for a pharmacy delivery service (*n =* 3) or enlisting assistance from friends/family (*n =* 4), six patients overcame forgetfulness by using pill boxes, setting alarms or strategically placing medications where they could not be overlooked, five patients overcame side effects through medication changes, and eight patients overcame the barrier of uncertainty as to why a medication had been prescribed through medication-education provided by a student.

Most students (*n =* 37) rated (from their perspective) the impact that MR had on their patients. Just over half of the students (*n =* 21) perceived the information they shared with their patient had a neutral or “informational only” effect; however, one student intervened during a life-threatening situation involving bleeding complications with warfarin. Three students believed that their intervention averted causing/aggravating organ dysfunction, and 12 students believed their interventions brought patients in line with acceptable standards of care.

## Discussion

### Significance to medical education

The medication reconciliation process is more complex than just comparing lists of medications. When performed appropriately, MR is a complex process requiring advanced skills that not only identifies the list of medications a patient is taking, but also involves clarifying and determining dosage/utilization/adherence, making necessary changes, and communicating this information effectively to both patients and other providers [10].

In this study, we embedded a MR/safety curriculum into a longitudinal PCMH course and taught students to apply a medication framework to assess medication appropriateness [8]. During the clinical years, students develop frameworks for approaching many aspects of patient care (e.g. performing patient histories, physical examinations), and we sought to provide trainees with a framework for approaching medication management as well. We believe that use of this framework enhanced our students’ self-described abilities and confidence to effectively manage medications and improved students’ performance on a MR clinical case. More importantly, students were able to take what they had learned in the classroom and apply it in clinical environments where they had a positive impact on patient care by identifying medication-related problems, educating patients about medications, and resolving patient barriers to medication adherence. As a result of our curricular design and data collection, we were able to demonstrate that we reached even the highest levels of Kirkpatrick’s four level model for judging the effectiveness of an educational initiative [11].

There is little doubt that the MR process benefits from multidisciplinary input. Yet, it is ultimately the physician’s responsibility to validate medication histories and formulate admission and discharge prescriptions. In academic medical centers and teaching hospitals, these duties are most often carried out by physicians-in-training (fellows, residents, or medical students) who are caring for vulnerable patients [10]. Yet, despite this, as a recent review demonstrates, very few medical schools place emphasis on MR in medical school curricula [10]. Furthermore, when MR topics have been included as curricular initiatives, the outcome measures have not necessarily been patient-centered. For example, van Zuilen and colleagues provided an online tutorial and classroom instruction to teach second year medical students how to take a medication history (including a MR segment) and used computer-cases and standardized patients to assess competencies. However, no comparison (e.g. pre- post- comparisons or a control group) was included, nor did students have opportunities to demonstrate proficiency with these skills with actual patients [12]. Similar to our PCMH curriculum, a “transitions” curriculum reported by Bradley et al. indicated that students’ self-reported confidence in performing MR and communicating medication information to patients improved after patient safety/discharge planning/health literacy topics were added to a 3rd year clerkship; however, in this curriculum there was no indication that students had opportunities to practice these skills with either real or standardized patients – thus questioning if either student behaviors or patient care was impacted by this new clerkship component [13].

Two additional reports in the literature describe MR curricular components that target medical students [14, 15]. Both of these involve inter-professional teams of medical and pharmacy students in the context of a transitions-in-care course. Similar to data we collected, students completed self-assessment questions about knowledge, confidence, and abilities. In addition, students had an opportunity to interact with one patient in a single post-hospital-discharge home-visit to assess factors relevant to safe discharge, which uncovered some medication-related problems. Most of the medication-related issues reported by these teams involved medication non-adherence and poor patient education [14, 15].

We believe that teaching MR in the context of a curricular structure such as the longitudinal PCMH course has several advantages over introducing the concept in traditional clerkships. These include multiple opportunities to practice a complex concept, longitudinal involvement of an inter-professional group of faculty instructors, and a strong emphasis placed on patient *relationships*. A key emphasis in our curriculum was practice. Students actively practiced MR in the classroom with their peers, on their own by completing MR clinical cases, as well as with all patients on their PCMH panels over the course of 9 months. The classroom-based activities (e.g. standardized patient; tools that were provided to assist students with MR) were designed specifically to facilitate students’ *critical thinking* about medication (in)appropriateness among patients, as opposed to making assumptions that computerized information regarding patient health (electronic medical records) is entirely complete or accurate [16].

We believe much of the success of the MR thread in the PCMH elective may also be attributed to the openness and honesty of the inter-disciplinary faculty in sharing personal experiences of medication prescribing mis-adventures. It is imperative that medical students not only hear about medication management concepts from a pharmacist’s perspective, but medical students must especially hear the significance of these same concepts reinforced by a physician colleague who speaks openly and honestly about the challenges of medication management. Interns and residents spend a substantial amount of time performing tasks that appear to be little more than administrative duties; MR may then, on the surface, appear to be little more than a mundane documentation task [16]. Therefore, having physicians promote MR as an *essential part of patient care,* as opposed to an added administrative burden, is imperative if we wish to develop positive medication management attitudes/behaviors among young trainees.

The formation of personal relationships between students and their patients is also a key factor underlying the impact that this MR thread had in our PCMH elective. For most students, there is a constant shift of rotations, institutions, and lack of long-term follow-up with patients. Trainees may view themselves as transient care providers [17]. For this reason, longitudinal clerkships are often considered to be superior to traditional clerkships in measures of patient and student satisfaction because they are better suited for patient-centered communication and care [18–20]. The personalized attention patients receive from students may increase patients’ perceptions of the quality of their care and empower them to be more engaged in self-management, including medication-related behaviors [21]. As such, longitudinal clerkships may be an ideal environment to introduce and reinforce medication management skills. In longitudinal curricular structures, students generally are given greater autonomy and have more opportunities to practice critical thinking skills while tackling progressively complex problems under direct preceptor supervision. Ultimately, longitudinal curricula that place emphasis on medication safety, may allow students to forge an easier transition into the role of a safe and effective prescriber.

Since only a subset of medical students at our institution will have longitudinal clerkship experiences, yet they all need to be able to prescribe medications safely and effectively, we have begun launching specific components of the MR curriculum into clerkships for *all* third year medical students at our institution. Furthermore, we are not teaching these concepts to medical students in silos, but we have also begun to include final year pharmacy students and second year nurse practitioner students into the medication management classroom activities. In our ongoing studies, we plan to evaluate the effects of such manipulations on our curriculum.

### Limitations

The chief limitation to our dataset is the small number of comparison students that we were able to recruit. Given the lack of power with our comparison group, we have been hesitant to place too much emphasis upon this group and reluctant to draw firm between-group conclusions. Nonetheless, our within-group pre- versus post- comparisons among enrolled students indicate that the curriculum elicited positive changes in student attitudes, behaviors, and critical thought regarding medication management in ways that had a discernible impact on patients. Although we asked students to report/classify the medication related problems that they uncovered, we did not ask them to reflect on potential causes or propose ways to avoid those problems in the future; such reflections may have been useful for addressing systems-based issues and preventing similar situations from occurring subsequently. In the future, it may be possible to implement mechanisms that seek to assess whether trainees continue to utilize these medication management skills into their residencies as well as to have longer periods of follow-up with patients to examine retention of new medication information/behavioral changes as long-term outcomes of the curriculum.

## Conclusions

Using a series of medication safety workshops, classroom discussions, medication reconciliation assignments, and simulation sessions, we addressed a number of previously-noted medication-related educational deficiencies. Students that participate in medication reconciliation/optimization curricular activities are better prepared to critically assess medications for safety and efficacy in medical practice.

## Abbreviations

IPE: Inter-professional education
MO: Medication optimization
MR: Medication reconciliation
MRPs: Medication-related problems
PCMH: Patient-centered medical home
PTA: Pharmacotherapeutic assessment
